# Molecular Mechanisms Linking ALS/FTD and Psychiatric Disorders, the Potential Effects of Lithium

**DOI:** 10.3389/fncel.2019.00450

**Published:** 2019-10-04

**Authors:** Fiona Limanaqi, Francesca Biagioni, Larisa Ryskalin, Carla L. Busceti, Francesco Fornai

**Affiliations:** ^1^Department of Translational Research and New Technologies in Medicine and Surgery, University of Pisa, Pisa, Italy; ^2^IRCCS Neuromed, Pozzilli, Italy

**Keywords:** frontotemporal dementia, amyotrophic lateral sclerosis, bipolar disorder, endoplasmic reticulum stress, unfolded protein response, autophagy, Munc13, RNA processing

## Abstract

Altered proteostasis, endoplasmic reticulum (ER) stress, abnormal unfolded protein response (UPR), mitochondrial dysfunction and autophagy impairment are interconnected events, which contribute to the pathogenesis of amyotrophic lateral sclerosis (ALS)/frontotemporal dementia (FTD). In recent years, the mood stabilizer lithium was shown to potentially modify ALS/FTD beyond mood disorder-related pathology. The effects of lithium are significant in ALS patients carrying genetic variations in the UNC13 presynaptic protein, which occur in ALS/FTD and psychiatric disorders as well. In the brain, lithium modulates a number of biochemical pathways involved in synaptic plasticity, proteostasis, and neuronal survival. By targeting UPR-related events, namely ER stress, excitotoxicity and autophagy dysfunction, lithium produces plastic effects. These are likely to relate to neuroprotection, which was postulated for mood and motor neuron disorders. In the present manuscript, we try to identify and discuss potential mechanisms through which lithium copes concomitantly with ER stress, UPR and autophagy dysfunctions related to UNC13 synaptic alterations and aberrant RNA and protein processing. This may serve as a paradigm to provide novel insights into the neurobiology of ALS/FTD featuring early psychiatric disturbances.

## Introduction

Altered functioning of the endoplasmic reticulum (ER) may lead to the accumulation of misfolded proteins, which cause ER stress. This maybe due to either genetic defects or post-translational modifications(Senft and Ronai, 2015). To maintain cell viability, ER stress recruits an adaptive reaction, the unfolded protein response (UPR). The UPR triggers a network of signaling cascades, which inhibit protein translation while up-regulating protein folding chaperones and cell-clearing systems. This occurs through the activation of specific stress sensors, which control protein folding, ER biogenesis, protein trafficking to and from ER, proteasome-dependent ER-associated degradation, autophagy, and exosome secretion, among others. When chronic and severe ER stress occurs, the UPR switches into UPR-mediated cell death via apoptotic signaling, providing a common link in various CNS disorders (Matus et al., 2008). Despite being distinct in nosography, mood disorders and amyotrophic lateral sclerosis (ALS) may intersect at various biochemical levels where the UPR is involved, and comorbidity between these disorders often occurs (Zucchi et al., 2019). In fact, neuropsychiatric conditions are overrepresented in ALS patients and psychiatric symptoms may even precede the onset of motor symptoms. In detail, a diagnosis of schizophrenia-like psychosis, bipolar disorder, depression, or anxiety, which is often associated with a first diagnosis of ALS within the following 1–5 years, is likely to specifically reflect the clinicopathological overlap of ALS with frontotemporal dementia (FTD, Velakoulis et al., 2009a, b; Byrne et al., 2013; Turner et al., 2016; O’Brien et al., 2017). Beyond proteinopathy, ALS/FTD features early synaptic alterations, which are reminiscent of those occurring in psychiatric disorders (Casas et al., 2016; Bradshaw and Korth, 2019). Besides protein aggregation and toxicity, the ER, UPR and cell-clearing pathways are implicated in synaptic plasticity (Hetz, 2012; Limanaqi et al., 2018, 2019b; Martínez et al., 2018). Thus, in ALS/FTD spectrum, extracellular stressors and/or genetic defects may trigger synaptic and neuronal dysfunctions through altered proteostasis, yielding concomitant psychiatric and neurological symptoms. In recent years, the mood stabilizer lithium has shown therapeutic potential in both mood disorder-related pathology and ALS/FTD (Fornai et al., 2008a, b; Pasquali et al., 2009; Chiu et al., 2013; Berk et al., 2017; Devanand et al., 2017; van Eijk et al., 2017; Machado-Vieira, 2018; for ALS see Table 1). As shown by a recent clinical study, the disease-modifying effects of lithium are remarkably significant in ALS patients carrying genetic variations in the UNC13 presynaptic protein, which are associated with FTD, ALS/FTD spectrum and psychiatric disorders as well (Diekstra et al., 2014; Lipstein et al., 2017; Nakamura et al., 2018). Furthermore, patients with psychiatric disorders receiving regular lithium treatment have a reduced prevalence of ALS and dementia (Kessing et al., 2008; Prosser and Fieve, 2016). In the brain, lithium modulates a number of biochemical systems which are involved in synaptic plasticity, proteostasis, and neuronal survival, and which are placed downstream of two main targets, namely glycogen synthase kinase 3beta (GSK3β) and mostly, phosphatidylinositol phosphatase pathway (Harwood, 2005; Pasquali et al., 2010a). By targeting UPR-related events, namely ER stress, excitotoxicity, and autophagy dysfunction either at the synapses or at cell bodies, lithium produces plastic effects on mood and motor activity, which may partly overlap with those responsible for neuroprotection. In the present mini-review, we discuss specific molecular events related to alterations in UNC13 as well as RNA and protein processing, which can be potentially modified by lithium. Lessons from the effects of lithium in relation with ER stress, UPR and autophagy may serve as a paradigm to disclose potential neurobiological mechanisms, and hopefully, therapeutic targets in ALS/FTD.

**TABLE 1:** Clinical and experimental studies reporting beneficial effects of lithium in ALS.

**ALS patients**	***In vivo* ALS models**	***In vitro* ALS models**
16 ALS patients received riluzole plus lithium, and 28 received riluzole only. At 15 months, all 16 patients treated with lithium and riluzole were alive, whereas 8 of 28 treated only with riluzole died (survival rate 100 vs. 71%). Lithium delayed disease progression in ALS patients as assessed by quantitative measurement of the muscle strength (by the MRC scale) and preservation of the pulmonary function (by FVC) (Fornai et al., 2008a).	Lithium enhances survival and motor function while protecting spinal cord motor neurons in G93A-SOD-1 mice from oxidative stress and Fas-related apoptosis. These effects are potentiated upon combined treatment with lithium and the anti-oxidant agent Neu2000 (Shin et al., 2007). Lithium delays the onset of disease symptoms prolonging the lifespan and decreasing the neurological deficit scores in G93A-SOD-1 mice while conferring neuroprotection through GSK3 inhibition in the brain and lumbar spinal cord. These effects are potentiated upon combined treatment with lithium and valproic acid (VPA, Feng et al., 2008). Lithium confers neuroprotection, delays disease onset and duration and augments the life span in G93A SOD-1 mice, through activation of autophagy, stimulation of mitochondriogenesis, and suppression of reactive astrogliosis (Fornai et al., 2008a).	Lithium pretreatment protects primary rat cerebellar granule against glutamate-induced excitotoxicity cells through GSK3 inhibition (Leng et al., 2008). Lithium protects mice primary motor neurons and organotypic chick embryo spinal cord neurons against kainic acid-induced excitotoxicity through GSK3b inhibition and activation of autophagy (Calderó et al., 2010; Fulceri et al., 2011).
The study enrolled 18 ALS patients to be compared with 31 ALS out-patients. Lithium and valproate co-treatment significantly increased ALS patients’ survival and exerted neuroprotection by increasing antioxidant defense markers assayed at baseline, and 5 and 9 months in plasma samples. The trial stopped after 21 months, due to the late adverse events of the treatment (Boll et al., 2014).	Lithium attenuates neurodegeneration in the brainstem (trigeminal, facial, ambiguous, and hypoglossal nuclei) of G93A SOD-1 mice while rescuing hypoglossal recurrent collaterals (Ferrucci et al., 2010). Lithium induces mitophagy and mitochondriogenesis to reverse the severe subcellular pathology, which occurs mostly within peripheral motor axons and muscles of G93A SOD-1 mice (Natale et al., 2015).	Lithium protects primary cultures of embryo rat motor neurons from neurotoxicity which is induced by cerebrospinal fluids (CSFs) from ALS patients (Yáñez et al., 2014).
Data from 3 randomized trials on 518 participants showed that although lithium does not improve overall 12-month survival rate in the general ALS population, in UNC13A carriers, it increases the 12-month survival probability from 40.1% to 69.7% (van Eijk et al., 2017).	Lithium suppresses the upregulation of Notch signaling and the postsynaptic protein Homer1b/c in the spinal cord of G93A SOD-1 mice to confer neuroprotection by increasing the Bcl-2/Bax ratio. These effects are potentiated upon combined treatment with lithium with VPA (Wang et al., 2015; Jiang et al., 2016).	Lithium suppresses the upregulation of Notch signaling and the postsynaptic protein Homer1b/c to confer cytoprotection in mtSOD1 (G93A) NSC34 cells (hybrid cell line of mouse neuroblastoma and embryonic spinal motor neurons) by increasing the Bcl-2/Bax ratio. These effects are potentiated upon combined treatment with VPA (Wang et al., 2015; Jiang et al., 2016).

## ER Stress, UPR and Autophagy in ALS/FTD and Bipolar Disorder

Endoplasmic reticulum stress and abnormal UPR play a central role in the pathogenesis of both psychiatric disease and ALS (Walker and Atkin, 2011; Cheng et al., 2014; Bengesser et al., 2016; Muneer and Shamsher Khan, 2019). Evidence for altered UPR in bipolar disorder stems from *ex vivo* studies documenting an abnormal response to ER stress-inducers. In detail, following stimulation with thapsigargin and tunicamycin, blood cells from bipolar patients show either unresponsive or reduced expression of the UPR markers p-eIF2α, GRP78, GRP94, XBP1, and CHOP (So et al., 2007; Hayashi et al., 2009; Pfaffenseller et al., 2014). Remarkably, changes in these markers predict lithium responsiveness in bipolar patients (Breen et al., 2016). This is not surprising since lithium may confer cytoprotection by recruiting these very same ER-stress related genes (Bown et al., 2000; Shao et al., 2006; Kakiuchi et al., 2009). Among bipolar patients, lithium was shown to be specifically effective in carriers with *XBP1*-116C allele, which has been identified as a risk factor for bipolar disorder (Masui et al., 2006).

In experimental ALS, ER stress increases the susceptibility of wild type SOD1 to aggregation (Medinas et al., 2018). Several UPR markers are increased in the spinal cord and blood of ALS patients and mouse models, with XBP1 activation representing an early pathological event in motor neuron disease (Ilieva et al., 2007; Atkin et al., 2008; Hetz et al., 2009; Ito et al., 2009; Matus et al., 2013; Montibeller and de Belleroche, 2018; Vats et al., 2018). As shown by *in vitro* studies, lithium may alleviate ER stress through GSK3β inhibition (Song et al., 2002; Takadera et al., 2007; Meares et al., 2008, 2011) and modulation of gene transcription via the PKC-GSK3β-AP-1 axis (Boyle et al., 1991; Manji et al., 2001; Hiroi et al., 2005).

Unfolded protein response activation is known to induce autophagy (Rashid et al., 2015; Yan et al., 2015), which counteracts ER stress via degradation of protein aggregates and organelles including damaged mitochondria and ER. Nonetheless, autophagy alterations occur in both ALS/FTD and bipolar disorder (Fornai et al., 2008a; Pasquali et al., 2009; Ferrucci et al., 2011; Malhi et al., 2013; Kim et al., 2017; Ramesh and Pandey, 2017; Bar-Yosef et al., 2019; Deng et al., 2019), which may be associated at least in part, with an abnormal UPR response. For instance, despite being IRE1/XBP1 pathway generally considered as an autophagy inducer, XBP1s loss promotes FoxO1-dependent autophagy conferring neuroprotection in neurons (Vidal et al., 2012). Again, deletion of XBPI in SOD1 transgenic mice produces a phenotype, which is resistant to developing ALS, and this is associated with autophagy activation (Hetz et al., 2009; Matus et al., 2009). It is remarkable that lithium rescues autophagy failure occurring in both ALS/FTD and bipolar disorder (Fornai et al., 2008a, b; Malhi et al., 2013; Toker and Agam, 2014; Merenlender-Wagner et al., 2015; Natale et al., 2015; Kim et al., 2017; Ryskalin et al., 2018). This suggests that lithium may counteract ER stress and abnormal UPR through autophagy induction. As proof of concept, *in vivo* lithium administration decreases ER stress-associated proteins GRP78, ATF-6, and CHOP while promoting the autophagy flux to protect motor neurons in the spinal cord (He et al., 2017; Tong et al., 2018).

## Munc13 Bridging Synaptic Alterations and ER Stress in ALS/FTD and Bipolar Disorder

Emerging evidence indicates a key role of the UPR in modulating synaptic function and connectivity. Altogether, ER proteostasis, UPR signaling, and cell clearing systems modulate behavior through intracellular pathways, which are involved in brain development and neuronal plasticity (Hetz, 2012; Limanaqi et al., 2018, 2019a,b; Martínez et al., 2018). In fact, the UPR in all its branches intermingles with the secretory pathway to finely tune the expression, synthesis, and folding of synaptic proteins. This occurs during their trafficking from ER, along with degradation by the proteasome and autophagy. Thus, UPR-related alterations may lead to synaptic remodeling and dysfunctions, which may occur independently of neurodegeneration (Hetz, 2012; Limanaqi et al., 2018, 2019a,b; Martínez et al., 2018). Detrimental changes in synaptic structure and function, namely synaptopathies, are considered as major contributors in psychiatric and neurological disorders (Lipstein et al., 2017; Limanaqi et al., 2018, 2019a,b; Ryskalin et al., 2018). This is best exemplified by alterations of Munc13, the mammalian ortholog of *C. elegans* unc13, which primes synaptic vesicles for exocytosis and regulates neurotransmitter release at presynaptic terminals and neuromuscular junctions. Gene variations/mutations in *UNC13* occur in psychiatric conditions including bipolar disorder, as well as in ALS and FTD (van Es et al., 2009; Diekstra et al., 2014; Nakamura et al., 2018; Placek et al., 2019). Some *UNC13* polymorphisms are associated with TDP-43 pathology underlying the ALS/FTD spectrum (Diekstra et al., 2014). In detail, *UNC13A* is associated with *in vivo* frontotemporal cortical atrophy, impaired cognitive performance, and greater burden of pTDP-43 pathological inclusions in sporadic ALS (Placek et al., 2019). Some variants in *UNC13A* are associated with increased disease prevalence and shorter survival in sporadic ALS patients (van Es et al., 2009; Diekstra et al., 2012; Yang et al., 2019). As recently described in a patient carrying a *de novo UNC13* mutation, even subtle changes in Unc13 structure can be deleterious for synaptic transmission, leading to concomitant psychiatric and neurological deficits (Lipstein et al., 2017).

As shown by a recent introspective clinical study, the effects of lithium among ALS patients are remarkable in *UNC13A* carriers, leading to a 69.7% increase in the 12-month survival rate (van Eijk et al., 2017). Therefore, in the present section we try to identify Munc13-related molecular events modified by lithium. In this context, it is fascinating that the regulatory domains of Munc13 are sensitive to lithium-dependent second messengers such as the phospholipase C (PLC)-inositol phosphate (IP3)/diacylglycerol (DAG, Brose et al., 1995).

### Ca^2+^ Signaling

The behavioral phenotype caused by a gain of function Munc13 was recently explained by the presynaptic Ca^2+^ influx via voltage-gated Ca^2+^ channels (VGCCs, Calloway et al., 2015; Lipstein et al., 2017). In detail, gene variations in Munc13 may affect its Ca^2+^ binding domain along with VGCC function leading an increased Ca^2+^ influx and synaptic vesicle exocytosis (Calloway et al., 2015). Dysregulation of Ca^2+^ homeostasis is prominent in both ALS and bipolar disorder (Machado-Vieira et al., 2011; Soeiro-de-Souza et al., 2012; Leal and Gomes, 2015; Machado-Vieira, 2018). Altered Ca^2+^ homeostasis generates ER stress, mitochondrial dysfunction, altered UPR along with impaired autophagy flux (Kim et al., 2002; Kawamata and Manfredi, 2010; Pasquali et al., 2010a, b; Fulceri et al., 2011; Cozzolino and Carrì, 2012; Soeiro-de-Souza et al., 2012; Filippi-Chiela et al., 2016). In detail, following abnormal stimulation of G-coupled receptors at the plasma membrane, PLC is recruited to produce DAG, a Munc13 binding substrate, and IP_3_. The latter binds to IP3 receptors to release Ca^2+^ from ER stores. At the same time, Ca^2+^ acts as a Munc13 binding substrate, thus leading to a vicious cycle of Munc13-dependent Ca^2+^ influx and abnormal neurotransmitter release. The release of Ca^2+^ from ER is buffered by mitochondria, which over time may be damaged leading to reactive oxygen species (ROS) production. These events lead to protein misfolding, which exacerbates ER stress while activating the apoptotic branch of the UPR (Decuypere et al., 2011; Prell et al., 2013; Leal and Gomes, 2015).

Patch-clamp recording and Ca^2+^ imaging can be affected by lithium selectively in those neurons from lithium-responder bipolar patients (Mertens et al., 2015), since lithium tones down Ca^2+^-related oxidative stress and mitochondrial damage (Wasserman et al., 2004; Pasquali et al., 2010a, b; Fulceri et al., 2011; Leal and Gomes, 2015; Kim H. K. et al., 2016). In keeping with this, the effects of lithium on mitochondrial function are remarkable in both ALS and mood disorders (Kubota et al., 2006; Toker and Agam, 2014; Natale et al., 2015). In fact, lithium reverses the abnormal behaviors resembling mood disorder in transgenic mice with mitochondrial dysfunctions (Kubota et al., 2006), and bipolar lithium-responders possess normal levels of constitutive mitochondrial genes compared with poor lithium responders (Stacey et al., 2018). Again, through stimulation of mitophagy and mitochondriogenesis lithium reverses mitochondrial alterations, which are associated with motor neuron degeneration and distal axon clogging in ALS experimental models (Natale et al., 2015; Ruffoli et al., 2015). These effects of lithium may relate to inhibition of either GSK3β or PIP2 pathway, which are both linked to synaptic transmission, Ca^2+^ dynamics, ER and mitochondrial function, and autophagy modulation (Schlecker et al., 2006; Pasquali et al., 2010a, b; Decuypere et al., 2011; Ringsevjen et al., 2019). For instance, via inhibiting IP3 turnover and GSK3b, lithium activates NRF2 (Castillo-Quan et al., 2016), which orchestrates the fine dynamics between autophagy/mitophagy and mitochondriogenesis (Palikaras et al., 2015; Ruffoli et al., 2015).

### Diacylglycerol, the Paradigm of Munc13 and PKC

Changes in DAG levels, which are produced by PLC at nerve terminals, are seminal to control the rate of neurotransmitter release through the activation of both Munc13 and protein kinase C (PKC, Nishizuka, 1992; Silinsky and Searl, 2003). Altered levels of DAG leading to abnormal activation of Munc13 and/or PKC converge into altering neurotransmitter release and Ca^2+^ influx. Alterations of PLC and DAG kinase are linked to mood disorders (Baum et al., 2008; Weber et al., 2011; Yang et al., 2016). Remarkably, PLC and DAG kinase may regulate mood through lithium-related pathways. In fact, ablation of PLC and/or DAG kinase produces a lithium-responsive mania-like behavior in animal models (Kakefuda et al., 2010; Isozaki et al., 2016; Yang et al., 2017).

**FIGURE 1:**
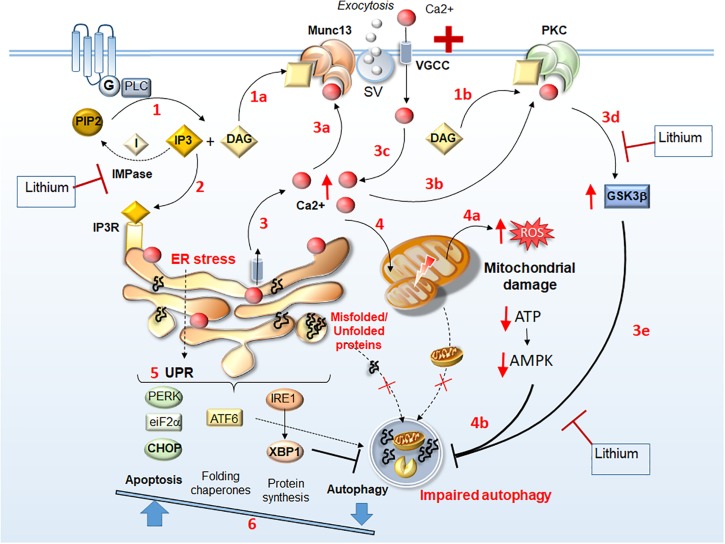
Identifying lithium-sensitive events related to Munc13 and PKC alterations. Following stimulation of G-coupled receptors, PLC is recruited to produce DAG and IP_3_ (1). DAG acts a binding substrate for both Munc13 and PKC activation (1a, 1b, respectively), while IP3 binds to IP3 receptors (IP3R) on the ER (2) to mobilize Ca^2+^ from internal stores (3). This leads to an increase of intracellular Ca^2+^, which in turn, acts as a binding substrate for both Munc13 and PKC (3a, 3b, respectively). Abnormal Munc13 and PKC activation lead to abnormal neurotransmitter release (synaptic vesicle, SV exocytosis), further Ca^2+^ influx (3c), PKC-dependent GSK3β activation (3d), and subsequent autophagy impairment (3e). Thus, intracellular Ca^2+^ levels increase dramatically leading to ER stress, accumulation of unfolded/misfolded proteins within the ER, and mitochondrial damage (4) along with ROS production, ATP depletion and AMPK downregulation (4a), which contribute to impairing autophagy (4b). The UPR attempts to restore homeostasis by increasing protein folding and degradation while inhibiting protein synthesis through PERK/eIF2a/CHOP, ATF6, and IRE1/XBP1 branches (5). Nonetheless, in conditions of a persistent ER stress, autophagy is also inhibited by XBP1, which shifts the UPR to apoptosis (6). Thus, misfolded/unfolded proteins and damaged mitochondria accumulate leading to a vicious cycle of chronic stress. Lithium may reverse these Munc13- and PKC-related molecular events either via GSK3β inhibition (steps 3d, 3e) or by reducing IP3 turnover through IMPase inhibition (steps 1, 2).

The role of PLC/DAG-dependent alterations related to Munc13 in ALS/FTD remains unexplored. Nonetheless, it is tempting to speculate that alterations in PLC/DAG signaling play a role, since increased expression of PLC occurs within motor neurons of SOD1-G93A ALS mice. On the other hand, PLC ablation increases survival and reduces nuclear alterations within motor neurons (Staats et al., 2013). Thus, PLC may contribute to excitotoxicity either by increasing IP3 and Ca^2+^ release from ER, or through DAG-dependent Ca^2+^ influx through Munc13 and PKC activation (Figure 1). Conversely, by reducing PIP_2_ – IP_3_/DAG levels, lithium is likely to reduce those intracellular alterations, which are due to the overlapping activities of PKC and Munc13 (Figure 1). Consistently with a reduced generation of DAG from PIP_2_ (Wang et al., 2001), lithium mitigates the expression and activity of PKC, which occurs in patients with mood disorders (Manji and Lenox, 1999; Hahn et al., 2005). Lithium also inhibits PKC translocation to the plasma membrane while reducing its interaction with the receptor for activated C-kinase-1 (RACKS-1), which is enhanced in post-mortem brains of bipolar patients (Wang and Friedman, 2001). Besides mood disorders, increased levels and activity of PKC are detected in the cervical spinal cord of ALS patients compared with controls (Lanius et al., 1995; Wagey et al., 1998). Increased PKC activity may affect neuronal viability and foster disease progression (Krieger et al., 1996). This is shown in ALS mice, where increased expression of PKC occurs, leading to downregulation of the chloride channel 1 (ClC-1) (Camerino et al., 2019). Since ClC-1 is key in sustaining neuromuscular junction and nerve integrity, its reduction leads to muscle hyper-excitability and impaired relaxation. Thus, PKC and Munc13 represent potential molecular targets in ALS (Varoqueaux et al., 2005; Veriepe et al., 2015; Camerino et al., 2019).

### Impaired Cell-Clearing Systems

Ablation of Munc13 in ALS mice bearing the *TDP-43-*A315T mutation is associated with decreased motor neuron degeneration compared with mice harboring A315T mutation alone (Veriepe et al., 2015). This is linked to abnormal Munc13-dependent exosome release, which may foster the exosome-mediated extracellular spreading of undigested TDP-43 (Veriepe et al., 2015). Again, this is related to autophagy impairment, which may spread prion-like proteins via exocytosis (Brundin et al., 2010), while lithium administration prevents the accumulation and spreading of prion-like proteins through induction of autophagy (Heiseke et al., 2009). Since proteasome and autophagy modulate neurotransmitter release and synaptic plasticity (Limanaqi et al., 2019a, b), a failure in the physiological turnover of Munc13 due to impaired cell-clearing pathways may lead to severe synaptic alterations.

## Dysfunctions of RNA and Protein Processing in ALS/FTD and Mood Disorders

Analysis of the genes and proteins at the heart of the continuum between ALS and FTD highlights a close connection between dysfunctions of RNA processing and autophagy as key events in disease pathophysiology (Thomas et al., 2013; Mandrioli et al., 2019). As support to such a functional convergence, a large amount of genes encoding proteins that are linked to ALS/FTD spectrum is involved in RNA metabolism and autophagy (Fornai et al., 2008b; Pasquali et al., 2010b; Mandrioli et al., 2019). This is best exemplified by mutations in *TARDBP*, *FUS*, and *C9ORF72*, which affect both global cellular RNA metabolism and autophagy. *TARDBP*, *FUS*, and *C9ORF72* encode proteins with prion-like disordered domains, and dipeptide repeat polymers, respectively. These undergo phase separation to form stress granules (SGs) involving the UPR-related translation initiation factors eIF2α and eIF3 (Colombrita et al., 2009; Ratti and Buratti, 2016; Boeynaems et al., 2017; Zhang et al., 2018; Mandrioli et al., 2019). Remarkably, structures being reminiscent of SG, which are composed of dipeptide repeat polymers co-localizing with ribosomal subunits and eIF3, were recently detected in the brain of c9ALS/FTD patients (Zhang et al., 2018). Although the role of SGs remains to be fully elucidated, *TARDBP*, *FUS*, and *C9ORF72* mutations are suggested to impair both protein translation and autophagy-dependent SG degradation (Lee, 2015; Chitiprolu et al., 2018; Mandrioli et al., 2019). Thus, when UPR is aberrant and autophagy flux is impaired, SGs may persist giving rise to potentially toxic proteinaceous inclusions (Ryu et al., 2014; Lee, 2015; Monahan et al., 2016; Dimasi et al., 2017; Deng et al., 2019). As such, autophagy inducers represent a potential therapeutic strategy against altered SG processing in ALS/FTD. In keeping with this, it is interesting to note that lithium may induce autophagy through eIF2α activation besides GSK3β inhibition (Relaño-Ginés et al., 2018). Even genes and proteins which are associated with bipolar disorder converge on UPR-related pathways controlling translation initiation and RNA processing (Carter, 2007; Darby et al., 2016; Laguesse and Ron, 2019). For instance, disrupted in schizophrenia 1 (DISC1) and neuregulin (NRG), which are implicated synaptic alterations and defective cytoskeleton-related organelle transport, induce eIF3-dependent SG assembly in response to environmental stressors (Ogawa et al., 2005; Kim J.A. et al., 2016). NRG2 also localizes to SGs, and depletion of NRG2 inhibits SG aggregation to promote cell survival during ER stress (Kim J.A. et al., 2016). Remarkably, alterations of DISC1 and NRG may also affect autophagy, since both of them are associated with upstream signaling pathways, which converge on the AKT-GSK3β/mTOR axis (Beaulieu, 2012; Ryskalin et al., 2018). NRG genetic variants are strongly associated with lithium responsiveness in mood disorders (Miranda et al., 2019); on the other hand, lithium normalizes the defective organelle transport caused by mutated DISC1 (Flores et al., 2011). In summary, these pieces of evidence suggest that abnormal UPR, altered RNA and protein processing and autophagy impairment may represent a lithium-sensitive molecular cascade implicated in the neurobiology of ALS/FTD.

## Concluding Remarks

Lithium remains the gold-standard therapeutic option for bipolar disorder. Despite years of inconclusive and disappointing results, lithium has been recently regarded as a potential neuroprotective drug in ALS, as shown by both translational and clinical studies. It is intriguing that the disease-modifying effects of lithium occur in a specific subpopulation of ALS patients bearing *UNC13* variants, which it turn, are linked to bipolar disorder and ALS/FTD spectrum.

Lithium-sensitive psychiatric disorders, such as bipolar disorder, depression and anxiety may often precede ALS/FTD, and lithium prophylaxis in mood disorders is associated with reduced prevalence of ALS and dementia. In the present mini-review, we discuss for the first time evidence suggesting that, at the molecular level lithium may target Unc13-related changes of synaptic activity, which produce concomitant neurological and psychiatric symptoms. The potential neuroprotective effects of lithium rely on the fact that it modulates several intracellular pathways involved in ER stress, Ca^2+^ toxicity, UPR, autophagy, and mitochondrial function. By rescuing the autophagy pathway, lithium may also target UPR-related dysfunctions of RNA and protein processing, which occur in bipolar disorder and most consistently, in ALS/FTD pathophysiology. Further studies elucidating the molecular mechanisms of action of lithium in relation with ER stress, UPR and autophagy, may disclose potential neurobiological mechanisms operating early in ALS/FTD and hopefully, preventive or therapeutic targets.

## Author Contributions

FL and FB drafted and wrote the manuscript, and contributed to the artwork. LR and CB made the literature research, manuscript editing, and contributed to the artwork. FF coordinator of the manuscript, he critically revised the manuscript for important intellectual content.

## Conflict of Interest

The authors declare that the research was conducted in the absence of any commercial or financial relationships that could be construed as a potential conflict of interest.
